# iManageMyHealth and iSupportMyPatients: mobile decision support and health management apps for cancer patients and their doctors

**DOI:** 10.3332/ecancer.2018.848

**Published:** 2018-07-11

**Authors:** Fatima Schera, Michael Schäfer, Anca Bucur, Jasper van Leeuwen, Eric Herve Ngantchjon, Norbert Graf, Haridimos Kondylakis, Lefteris Koumakis, Kostas Marias, Stephan Kiefer

**Affiliations:** 1 Fraunhofer Institute Biomedical Engineering, 66280 Sulzbach, Germany; 2 Philips Research, High Tech Campus 34, 5656 AE Eindhoven, The Netherlands; 3 Department of Pediatric Oncology and Hematology, Universität des Saarlandes, Campus Homburg, 66421 Homburg, Germany; 4 Foundation for Research and Technology Hellas, Institute of Computer Science, N Plastira 100, GR-70013 Heraklion, Greece

**Keywords:** clinical decision support, predictive models, patient guidance services, self-management

## Abstract

Clinical decision support systems can play a crucial role in healthcare delivery as they promise to improve health outcomes and patient safety, reduce medical errors and costs and contribute to patient satisfaction. Used in an optimal way, they increase the quality of healthcare by proposing the right information and intervention to the right person at the right time in the healthcare delivery process.

This paper reports on a specific approach to integrated clinical decision support and patient guidance in the cancer domain as proposed by the H2020 iManageCancer project. This project aims at facilitating efficient self-management and management of cancer according to the latest available clinical knowledge and the local healthcare delivery model, supporting patients and their healthcare providers in making informed decisions on treatment choices and in managing the side effects of their therapy. The iManageCancer platform is a comprehensive platform of interconnected mobile tools to empower cancer patients and to support them in the management of their disease in collaboration with their doctors. The backbone of the iManageCancer platform comprises a personal health record and the central decision support unit (CDSU). The latter offers dedicated services to the end users in combination with the apps iManageMyHealth and iSupportMyPatients. The CDSU itself is composed of the so-called Care Flow Engine (CFE) and the model repository framework (MRF). The CFE executes personalised and workflow oriented formal disease management diagrams (Care Flows). In decision points of such a Care Flow, rules that operate on actual health information of the patient decide on the treatment path that the system follows. Alternatively, the system can also invoke a predictive model of the MRF to proceed with the best treatment path in the diagram. Care Flow diagrams are designed by clinical experts with a specific graphical tool that also deploys these diagrams as executable workflows in the CFE following the Business Process Model and Notation (BPMN) standard. They are exposed as services that patients or their doctors can use in their apps in order to manage certain aspects of the cancer disease like pain, fatigue or the monitoring of chemotherapies at home. The mHealth platform for cancer patients is currently being assessed in clinical pilots in Italy and Germany and in several end-user workshops.

## Introduction

The prevalence of chronic diseases is rising. This is true for children, adolescents and young adults as well as the elderly [1]. Most cancers can be regarded as chronic diseases, as their treatment lasts for a long period of time and multiple follow-ups are needed to detect as early as possible a relapse or late effects of treatment.

Clinical decision support systems (CDSSs) are important and necessary because they can play a crucial role in healthcare delivery as they promise to improve health outcomes and patient safety, avoid adverse events, reduce medical errors and costs and contribute to patient satisfaction. Properly integrated CDSSs can assist clinicians in preventing adverse drug reactions, reducing inappropriate drug dosing, and reinforcing the use of effective prophylactic measures [2–5]. Used in an optimal way they increase the quality of healthcare by proposing the right information and intervention to the right person at the right time in the healthcare delivery process. CDSSs also have the potential to reduce the knowledge gap between clinical research and practice, as they allow for an efficient transfer of the latest clinical research findings implemented as clinical decision models to the clinical care setting. A typical CDSS is a computerised system that uses case-based reasoning to assist clinicians in assessing disease status, in making a diagnosis, in selecting appropriate therapy or in making other clinical decisions. Typically, a CDSS leverages actual clinical data of the patient in different tools such as clinical guideline, diagnostic support and treatment selection support, which are provided to clinical users, patients and/or other caregivers at various points in time for meaningful support in decision making.

CDSSs have been used for different purposes ranging from diagnosis, preventive care and therapy to monitoring and follow-up. The most common workflow-integrated decision support systems of the past decades are the Arden Syntax (http://www.hl7.org/special/Committees/arden/index.cfm), PROforma, Asbru, PRODIGY, SAGE, SEBASTIAN, GLARE described on the OpenClinical platform (http://www.openclinical.org) as well as the CDSS developed in the EU projects CHRONIOUS [6] and d-LIVER [7]. SAGE [8, 9] and SEBASTIAN [10] are the most prominent examples of CDSS systems based on service models.

More specifically, for the oncology domain, there are several CDSSs, such as eviti (http://www.eviti.com/), Proventy (http://proventys.com/), Adjuvant (http://www.adjuvantonline.com/), MedSolution (http://www.medsolutions.com/) and Arezzo Optimal Pathways (http://www.infermed.com/en/Clinical-Decision-Support/Arezzo-Pathways-Solutions.aspx). Most of these CDSSs support decision-making needs of health professionals by providing real-time access to protocols and guidelines for patient’s specific treatment decisions for oncology treatments.

A specific approach to integrated clinical decision support and patient guidance in the cancer domain is taken by the H2020 iManageCancer project (http://imanagecancer.eu/about), a research collaboration of nine partners with clinical, academic and industrial background from five European countries [11]. It aims at facilitating efficient self-management and management of cancer according to the latest available clinical knowledge, actual patient data, and the local healthcare delivery model, supporting patients and their healthcare providers in making informed decisions on treatment choices and in managing side effects of their cancer therapy.

## Decision support approach in iManageCancer

iManageCancer provides cancer patients and their physicians with a comprehensive platform of interconnected mobile tools to empower patients and to support them in the management of their disease in collaboration with their doctors. The back end of the iManageCancer platform contains an intelligent personal health record (iPHR) application [12] and also comprises the central decision support unit (CDSU). The CDSU offers dedicated disease management services to the end users in combination with the apps iManageMyHealth and iSupportMyPatients.

The main idea behind the developed CDSS is that domain experts design so-called care flow diagrams, formal disease management workflows [13], based on clinical guidelines, knowledge on care pathways and an organisational model for integrated healthcare with the patient as the co-manager of his health in the centre of it. We use the term ‘care flow’ here in the sense that our care flow diagrams describe the management of certain aspects of the disease of the patient in an outpatient setting or, more specifically, in a mHealth setting. For the formalisation of care flows and their integration in our system platform, we apply business process modelling following the Business Process Model and Notation (BPMN) 2.0. For executing care flows, we use the open source platform Activiti (https://www.activiti.org/).

**Figure 1.**
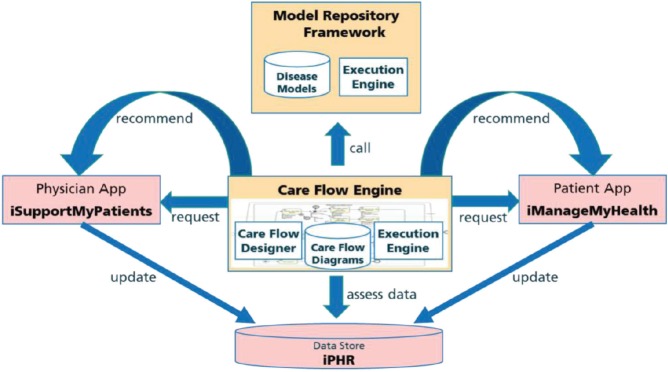
Care flow driven CDSU in iManageCancer platform.

A care flow is represented as a complex formal process diagram with integrated predictive models from a model repository framework (MRF). These care flow diagrams are personalised for a specific patient in individual care flow plans and executed by the CDSU of the iManageCancer Platform, in the so-called Care Flow Engine (CFE). The CFE guides the patient but optionally also the healthcare team through the management of the disease and related co-morbidities by issuing tasks and recommendations to the patient and to the different members of the healthcare team and by controlling the execution of the care flow plan based on the results of tasks and monitored health status of the patient. In the design phase of the care flow plan, clinical knowledge is modelled as a set of rules that control the execution of the plan. In decision points of such care flows, rules apply which operate on the actual health information of the patient and decide on the treatment path that the system follows. Alternatively, the system can also invoke a predictive model of the MRF to proceed with the best treatment path in the diagram. The interaction of these components is shown in Figure 1.

For designing meaningful care flow diagrams, the service-oriented iManageCancer platform provides a high degree of automation. Care flows can incorporate and leverage other services of the platform. The two apps iManageMyHealth and iSupportMyPatients include a client for using the care flows offered by the CFE and the related services for their execution (Figure 1). These apps download and process tasks for the patient or the doctor issued by the CFE. Health assessment questionnaires and measurement tasks represent fundamental types of tasks in the care flow diagrams. They provide a possibility to collect actual information on the patient’s current condition by the patient himself or his doctors. Advice is given by issuing information tasks or by linking to personalised information from high-quality public internet resources on cancer.

In this way, a flexible management of aspects of the patient’s disease is achieved, adapted to his actual conditions and clinical symptoms, together with a strict and clear integration among specialists, therapists and doctors, according to formal plans of the healthcare processes.

A set of care flow diagrams is modelled in iManageCancer by clinical experts for different aspects of cancer management, with a focus on those aspects of the disease that can be managed by the patient him/herself. In this way, passive and active decision support, reminders, questionnaires and related guidance can be incorporated in the process diagram. Besides data collected by the iManageMyHealth app, additional data are available and exploited by the CFE. Those data are collected within the iPHR system by the patients and further integrated and semantically uplifted [14, 15].

## Client services apps for patients and health professionals

Patients and their physicians who registered on the iManageCancer platform can utilise the management services provided by the CFE with the help of two individual apps.

The iManageMyHealth app for the patient downloads and processes the individual Care Flow tasks issued for himself or herself by the CFE. Tasks for patients are typically health enquiries and measurement tasks but also information providing tasks with the recommendations obtained from the CFE. Results are sent back to the CFE for further assessment and control. Similarly, the iSupportMyPatients app for physicians downloads and processes tasks for the patient’s physician. The both apps receive also notifications from the CFE about tasks assigned to health professionals or patients.

In addition to the management support for patients, the iManageMyHealth app also implements drug management and safety, recording of vital signs and blood parameters as well as management of paper-based clinical documents.

**Figure 2.**
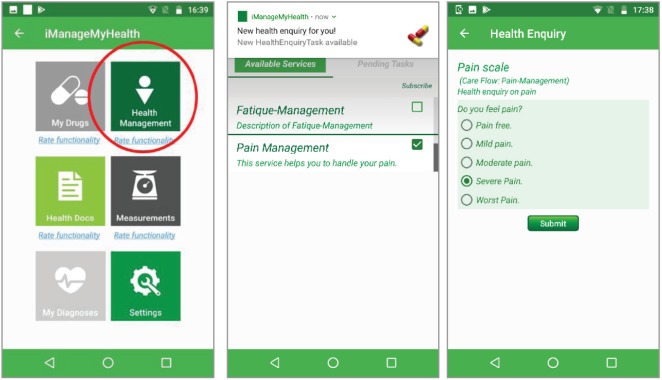
Care flow diagrams in the iManageMyHealth app. The patient can subscribe to these services or can unsubscribe from a care flow. A message about a pending task of the care flow for pain management is received on the smartphone. When the user touches the message, the system forwards him to a pain scale.

Figure 2 shows the iManageMyHealth app module to view and to manage the Care Flows and tasks received from the CFE. The patient can subscribe to specific care flows or unsubscribe from the already subscribed ones.

**Figure 3.**
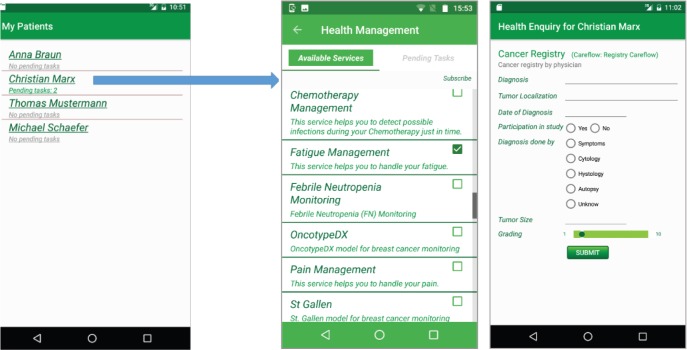
Care flow diagrams in the iSupportMyPatients app. The physician can subscribe to these services for a specific patient or he can unsubscribe from a care flow for this patient.

The app iSupportMyPatients for physicians, shown in Figure 3, receives the list of available care flows. Then, it visualises tasks from the CFE for all patients who enabled data sharing in their iPHR with their respective physician. The physician can see in the list of his patients the number of currently pending tasks that he shall execute. After selecting a patient or his pending tasks, the physician is forwarded to a page for managing care flows and tasks.

## Care flows

To show the potential benefits of our CDSU, we have implemented a set of diverse models in several management scenarios for patients and their doctors, represented as Care Flows:

- For health professionals: Care flows for chemotherapy management (febrile neutropenia (FN) care flow) and for breast cancer therapy assistance (St. Gallen—OncotypeDX Care Flow)
- For patients: Care flows to support patients in managing pain and fatigue

### Example febrile neutropenia care flow for chemotherapy management

Cytotoxic chemotherapeutic agents, radiotherapy and the tumour itself can result in myelosuppression and damage to barriers, which protect against infection. Severe neutropenic complications reduce the patients’ quality of life, lead to hospitalisation and increase their risk of mortality. Without swift intervention, infections in patients with neutropenia may ultimately lead to death.

The so-called Multinational Association of Supportive Care in Cancer (MASCC) model used in this care flow targets patients prior to chemotherapy treatment not just those who already have a febrile episode. The model stratifies patients and identifies those at higher risk of having an FN episode in the first cycle of chemotherapy treatment. Preventative interventions and additional resources can then be targeted at those high-risk patients with the aim to reduce FN emergency admissions. The model uses readily available clinical input risk factors and is transparent in the contribution of those factors in delivery of the overall FN risk level.

The intended end users of this scenario are clinicians (oncologists and nurses) and patients. Initially, the health professional will be asked to fill in a questionnaire with the MASCC model questions for his patient. The CDSU calculates a risk index. The health professional and the patient are informed about the risk score of the patient. The patient receives recommended information for handling this particular situation. Furthermore, the patient is asked to perform measurements using medical devices (e.g. for body temperature) or report symptoms and side effects according to the treatment. If the entered values are critical, the patient will get a recommendation (e.g. an advice to visit his doctor). His doctor is also informed about these recommendations.

### Pain management

An effective pain therapy is fundamental for the quality of life of many cancer patients. Not every cancer patient suffers from pain but those who do can be relieved from pain with modern therapies. The sensation of pain is subjective and electronic tools are expected to support the self-management of pain. Therefore, this scenario is motivated at preserving the patients’ quality of life through pain release, increasing patients’ well-being and efficacy and thus promoting the therapeutic success, as well as increasing information on patients’ pain perception for the attending physicians. In particular, it focuses on preventing patients from enduring pain and avoiding the intake of pain medication.

### Fatigue management

This care flow aims to reach the best treatment and handling of chronic fatigue in cancer (‘tumour fatigue’ caused by cancer disease, or accessory symptom caused by drug side effects, associated cancer symptoms like anaemia, mental stress, etc.). It supports the patients to budget their own energy and to avoid that they overtax themselves as well as to challenge them for improving their situation. Health professionals can work with patients with cancer to develop an activity/rest program based on an assessment of the patients’ fatigue patterns that allows the best use of the individual’s energy. Any changes in daily routine require additional energy expenditure. Individuals with cancer are advised on setting priorities and maintaining a reasonable schedule.

## Care Flow Engine

The CFE represents a care flow-driven web-based approach to decision support that guides patients and doctors with their end devices and corresponding applications through the care pathway. In addition, it supports them in decision making and management by providing corresponding high quality and precise information. Using Google Cloud Messaging Services, CFE sends notifications to patients’ smartphones to perform tasks with their app *iManageMyHealth* or similarly to health professionals for their app *iSupportMyPatients*. These tasks are provided to the apps via representational state transfer services in JavaScript object notation (JSON) format interpreted appropriately by the user interfaces of the apps. The results of the executed tasks of such a care flow (e.g. measurement and health enquiry values) are stored in a local database as well as in the patients’ health record on the iManageCancer platform.

**Figure 4.**
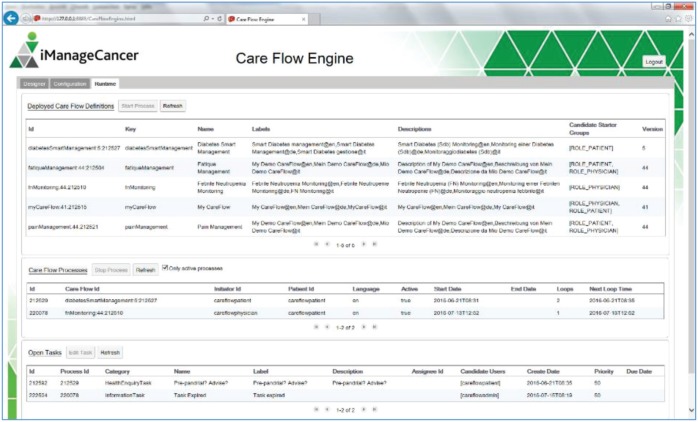
GUI of the CFE.

**Figure 5.**
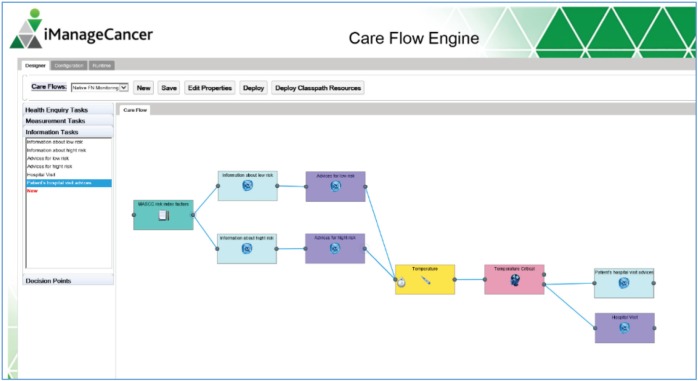
The care flow designer showing an example of a care flow diagram. The diagram is composed of different types of tasks (health enquiries, measurement and information tasks) and decision points represented as graphical elements available in the toolbar on the left.

**Figure 6.**
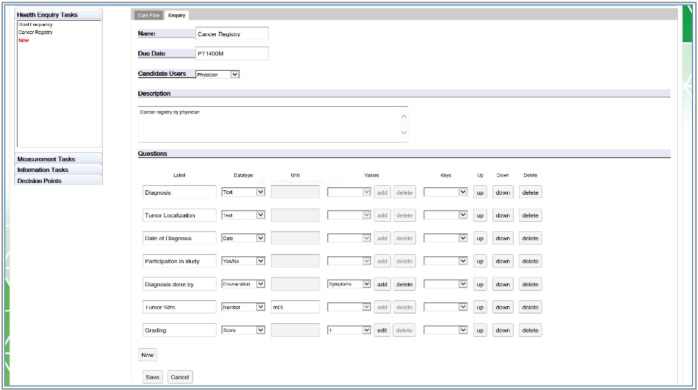
An example in the Editor creating a health enquiry task with all supported question item tasks.

**Figure 7.**
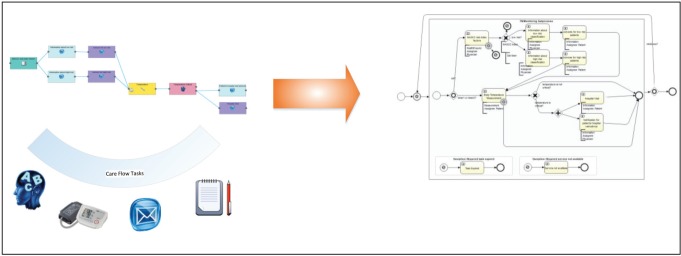
Transformation of the designed care flow in an object model following the BPMN 2.0.

The CFE user interface shown in Figures 4–7 contains an editor to create care flow diagrams (Care Flow Designer) and a dashboard to manage and monitor the available care flows and executed care flow processes for individual patients (Runtime). It is also possible to simulate executing processes and tasks in the runtime environment. Services used by the CFE can be configured using the provided graphical user interface (GUI).

An embedded generator transforms the designed care flow diagram into a deployable and executable Activiti BPMN object model. The graphical care flow is encoded in JSON.

## Model repository framework and provided models

There are several challenges that exist in the design, development and implementation of a CDSS which impede its successful employment in the healthcare domain. Some of these challenges include: how to intelligently manage patient’s clinical data, how to mine large clinical database to create new CDSSs, how to create an architecture and to define standard interfaces for sharing clinical decision support modules and how to efficiently disseminate new clinical decision modules and keep them up to date.

To address the above-mentioned issues, we propose a flexible model-based approach that enables decision support applications to cope with the high rate of growth of medical knowledge as presented in [16]. The proposed MRF enables the development of decision support tools that can easily integrate a large variety of multi-scale models providing flexibility as well as scalability and facilitates connecting the models with the required (patient) data. Clinical models can be easily integrated with the proposed framework and existing models can be easily upgraded and extended, therefore allowing the framework to deal with the high rate of changes in medical knowledge. Furthermore, these clinical models can be independently validated and gradually added to the clinical decision support tools for applications in patient care.

A prototype [16] of MRF is used to develop and deploy an implementation that combines community-developed models described in the literature (e.g. the St. Gallen stratification for early breast cancer) and knowledge models from research (e.g. Oncosimulator [17], microRNA model [18]) in the context of the p-Medicine project (www.p-medicine.eu) in the oncology domain.

**Figure 8.**
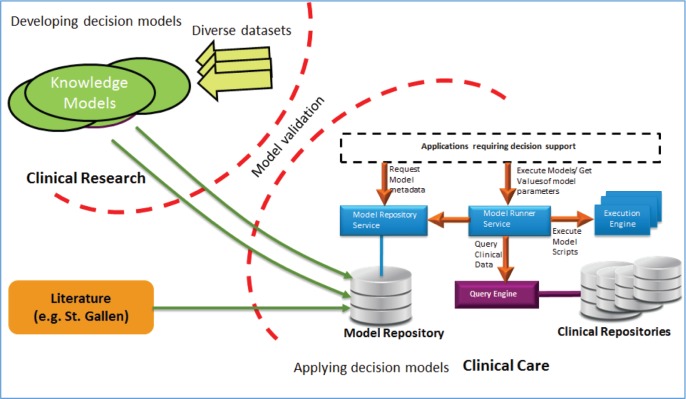
MRF of the CDSU of the iManageCancer platform.

The MRF and its underlying solution for storage, management and execution of models is shown in Figure 8: validated knowledge models from the research can be easily added to the model repository and can be further used for the clinical decision support in the clinical care by using several services provided in the framework. The MRF framework can also be used for the continuous validation of existing models on new data.

In the context of the iManageCancer project, the MRF modules provide support for the clinicians to understand the medical conditions and various treatment options for their patient, to better understand their diagnosis and follow a plan of care. In collaboration with CFE and iPHR, the MRF can be used to provide decision support by the healthcare professionals for patient empowerment.

The aim of the model repository is to store predictive models. The added models represent the validated knowledge from the research, and they can also be used by the MRF for continuous validation on new data.

Several established predictive models in the cancer domain have already been included in the model repository:

- **St. Gallen** (http://www.oncoconferences.ch/mm/Consensus_SG-2013.pdf)—a model proposing treatment recommendation for patients suffering from early breast cancer. It uses as input the following parameters: *Estrogen receptor (ER), Human epidermal growth factor receptor 2 (HER2), protein encoded by the MKI67 gene (Ki-67), Progesterone receptor (PgR).*
- **MASCC**—an established predictive model in the cancer domain for stratifying patients into low and high-risk groups with respect to serious adverse event(s) such as FN. It is envisioned that following appropriate clinical validation, the risk score(s) produced should improve the clinician’s ability to intervene before such an event occurs, as well as increase patient awareness with respect to their disease state. It takes as input the following parameters: *burden of illness, hypotension, pulmonary disease, solid tumour or fungal infection, outpatient, dehydration and age*; and it returns a risk score. The MASCC risk index can accurately identify patients with FN that have a low risk for complications. It can be used to select patients for more convenient or cost-effective therapies.
- **Wilms tumour**—a model that can based on his/her microRNA expression data, determine if the patient has the phenotype of a Wilms tumour or not.
- **BRC (breast cancer), intermittent bevacizumab prediction**—predicting the effect of a user-specified intermittent bevacizumab monotherapy scheme to a specific breast tumour.
- **BRC (breast cancer): bevacizumab comparison**—comparing the treatment outcomes while applying fractionated versions (total amount of drug spread out over total treatment period) of an original bevacizumab monotherapy scheme to a specific breast tumour.
- **Vincristine-Actinomycin**—predicting the effect of pre-operative chemotherapy with Actinomycin and Vincristine on Wilm’s tumour in individual patients.
- **OncotypeDX**—a model for scoring results of the OncotypeDX laboratory test. It predicts the recurrence of women breast cancer at early stage. It is based on 21 genes in order to estimate the probability of the benefit of chemotherapy; the scoring is a value between 0 and 100, and we use the following threshold: High (scoring > 18) and Low (scoring < 18)
- **Cardiac function**—a predictive model for calculating status of laboratory test values. It predicts the cardiac function of a patient as normal or abnormal.

**Figure 9.**
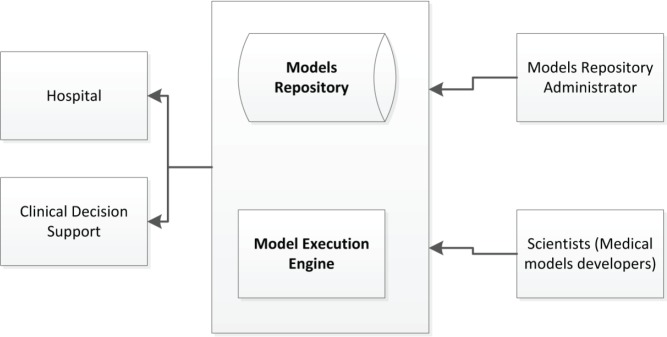
Model repository and model execution engine.

Users of the MRF can use the models utilising the corresponding available web services for model management (e.g. create, read, update, delete) and execution on the existing patient data as shown in Figure 9.

The model execution engine is a service providing support for execution of the predictive models. Depending on the framework and language used to build the predictive model, different engines can be specified (e.g. Jess, Drools, Java, Python, R).

To protect the access to the service, OAuth2 (https://oauth.net/2/) is used for authorisation. Patient data are not stored in the model repository, they are only provided as input for the models.

## Conclusions

With the CDSU, a powerful clinical decision support framework has been provided and integrated in the iManageCancer platform to support patients and their physicians in the management of their cancer. Implemented management services offered by the framework focus on chemotherapies and their side effects. The adopted service-oriented architecture and the corresponding modular approach allows adding new clinical models and care flows on the fly and updating existing ones. However, this dynamic nature of the system raises new challenges from the regulatory perspective, requiring a thorough risk analysis before new models and care flows are deployed in the system. Models need to be validated before they can be used as part of a care flow diagram in the clinical routine. As part of the back end of the iManageCancer platform, the CFE offers its services to patients and clinicians based on two sophisticated client apps: iManageMyHealth and iSupportMyPatients. The apps need to be online to use these services. The iManageCancer platform with its various tools is currently being evaluated in two clinical pilots with adult and paediatric cancer patients in Italy and Germany. Obviously, the developed clinical decision support framework is also applicable to other chronic diseases and their mobile management.

## Conflicts of interest

The authors have declared that no competing interests exist.
